# Inheritance of Fruit Red-Flesh Patterns in Peach

**DOI:** 10.3390/plants12020394

**Published:** 2023-01-14

**Authors:** Nathalia Zaracho, Gemma Reig, Naveen Kalluri, Pere Arús, Iban Eduardo

**Affiliations:** 1 Centre for Research in Agricultural Genomics (CRAG) CSIC-IRTA-UAB-UB, Campus UAB, Edifici CRAG, Cerdanyola del Vallès (Bellaterra), 08193 Barcelona, Spain; 2 Institut de Recerca i Tecnologia Agroalimentaria (IRTA) Fruitcentre, Programa Fructicultura, PCiTAL, Parc Gardeny, 25003 Lleida, Spain; 3 Institut de Recerca i Tecnologia Agroalimentaria (IRTA), Campus UAB, Edifici CRAG, Cerdanyola del Vallès (Bellaterra), 08193 Barcelona, Spain

**Keywords:** *Prunus*, anthocyanins, QTLs, marker-assisted breeding, fruit quality

## Abstract

Fruit color is an important trait in peach from the point of view of consumer preference, nutritional content, and diversification of fruit typologies. Several genes and phenotypes have been described for peach flesh and skin color, and although peach color knowledge has increased in the last few years, some fruit color patterns observed in peach breeding programs have not been carefully described. In this work, we first describe some peach mesocarp color patterns that have not yet been described in a collection of commercial peach cultivars, and we also study the genetic inheritance of the red dots present in the flesh (RDF) and red color around the stone (CAS) in several intra- and interspecific segregating populations for both traits. For RDF, we identified a QTL at the beginning of G5 in two intraspecific populations, and for CAS we identified a major QTL in G4 in both an intraspecific and an interspecific population between almond and peach. Finally, we discuss the interaction between these QTLs and some other genes previously identified in peach, such as dominant blood flesh (*DBF*), color around the stone (*Cs*), subacid (*D*) and the maturity date (*MD*), and the implications for peach breeding. The results obtained here will help peach germplasm curators and breeders to better characterize their plant materials and to develop an integrated system of molecular markers to select these traits.

## 1. Introduction

Peach, *Prunus persica* (L.) Batsch, is an economically important temperate fruit tree and one of the model species for the Rosaceae [1]. Peach breeding is a very active field, and many new cultivars are released each year [2]. Due to its long intergenerational period and the largeness of plants, peach breeding is also a resource-consuming activity, where strategies based on molecular markers can greatly improve its efficiency. Recently, molecular markers linked to major genes were described and are currently being used in peach breeding programs [3,4,5].

Fruit flesh color is an important trait for many species, including peach. According to the IPCGR peach descriptors [6], cultivars can be classified into eight flesh color types (greenish white, snow white, cream white, greenish yellow, light yellow, yellow, orange yellow, and orange). In addition, based on the anthocyanin coloration of the flesh, they can also be classified into seven categories: absent, weak, only under the skin, under the skin and around the stone, only around the stone, faint in the whole flesh, and intense in the whole flesh [6]. Most commercial peach cultivars are classified as white or yellow flesh, but in the last few years peach breeders have dedicated part of their efforts to obtain red (or blood) flesh cultivars to further diversify the fruit typologies available and because red-flesh peaches present high anthocyanin contents that promote human health [7].

Several genes affecting peach flesh color have been identified and molecular markers have been described in their neighborhood. The first one is the *Y* gene, responsible for yellow vs. white flesh. This gene was first described by Connors [8] and later mapped in linkage group 1 (G1) by Bliss et al. [9]. The gene responsible for the trait, a carotenoid cleavage dioxygenase (CCD4), has been described, and several mutations on this gene producing the same yellow flesh phenotype were identified [10,11]. The first gene related to peach blood flesh was discovered by Werner et al. [12] in different progenies from the red-fleshed cultivar Harrow Blood. Homozygous individuals for the recessive allele of this gene (*bf* for blood flesh) produced the colored fruit phenotype and the gene was mapped at the beginning of G4 by Gillen and Bliss [13]. A second gene producing peach red flesh is the dominant blood flesh (*DBF*), identified in the Chinese peach cultivar Wu Yue Xian and mapped at the beginning of G5 [14]. A third gene producing red flesh has been described in an interspecific peach by almond population [15]. This gene, called *DBF2*, mapped at the end of G1, with the dominant *DBF2* allele producing the red flesh phenotype. Another gene related to the peach flesh anthocyanin content described is the color around the stone (*Cs*), where the presence of the dominant allele results in a colored phenotype. *Cs* was segregated in a population derived from the cross Akame × Juseito and was mapped in G3 [16,17]. The *Cs* gene position was later confirmed using association analysis using whole-genome association studies (GWAS), although no diagnostic marker explaining the trait for all cultivars was identified [18].

In addition to these genes, other patterns of anthocyanins are often observed in the fruit of different peach cultivars. Our objectives in this paper are: (i) to describe some peach mesocarp color patterns that have not yet been described in a collection of peach commercial cultivars, and (ii) to contribute to the understanding of the genetic basis or other factors that may be involved in the expression of peach mesocarp red dots or the red color around the stone.

## 2. Results

### 2.1. Description of Peach Flesh Color Patterns Identified in the IRTA Peach Cultivar Collection

**Figure 1:**
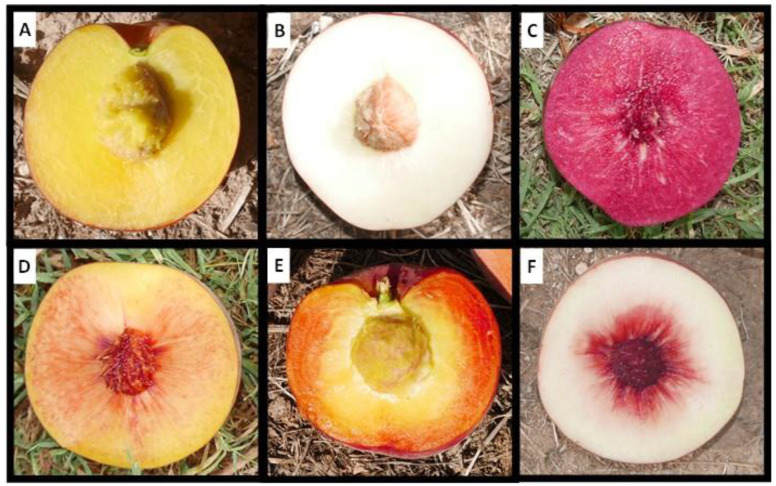
Image of the different flesh color phenotypes identified in the IRTA peach commercial cultivar collection. (**A**) Yellow flesh (Noracila). (**B**) White flesh (Nectarnovala). (**C**) Red flesh (Diablotina). (**D**) Red dots in the flesh (RDF) (Crispsol). (**E**) Red dots under the skin (RDS) (Britney Lane). (**F**) Red color around the stone (CAS) (Lucius).

**Figure 2:**
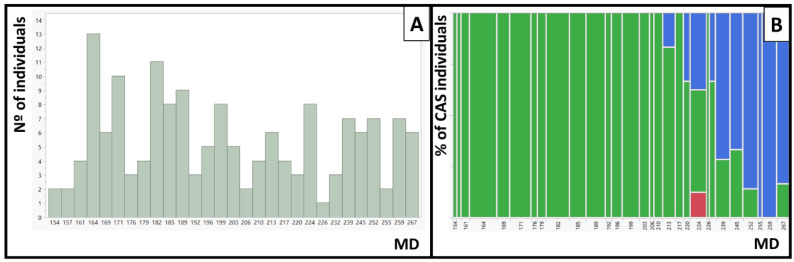
Maturity date (MD) and red color around the stone (CAS) in IRTA’s peach cultivar collection. (**A**) Distribution of the MD in Julian days. (**B**) Contingency table between CAS and MD. In green, percentage of individuals with no CAS, and in blue, percentage of individuals with CAS. In red, the Diablotina cultivar that was not phenotyped for CAS.

The different flesh color phenotypes identified in the IRTA’s peach commercial cultivar collection include yellow flesh, white flesh, red flesh, red dots in the flesh (RDF), red dots under the skin (RDS) and red color around the stone (CAS) and can be observed in Figure 1. The phenotypes of the 159 cultivars are presented in Table S1. In total there were 78 cultivars with yellow flesh, 80 with white flesh and one with red flesh. Ninety-one did not present red dots in the flesh and 67 did. Independently of the flesh color, 21 cultivars presented RDS and 35 CAS. The collection includes cultivars whose maturity date (MD) goes from the beginning of June until the end of September, covering the whole peach season harvest under Mediterranean conditions. While we found individuals with RDF and RDS along the whole season, cultivars presenting CAS were only identified at the end of the season, NETIX30 being the earliest one and ripening on 1 August (Figure 2).

### 2.2. QTL Analysis of RDF (Red Dots in the Flesh) in Bb × Nl and in SDF2

**Figure 3:**
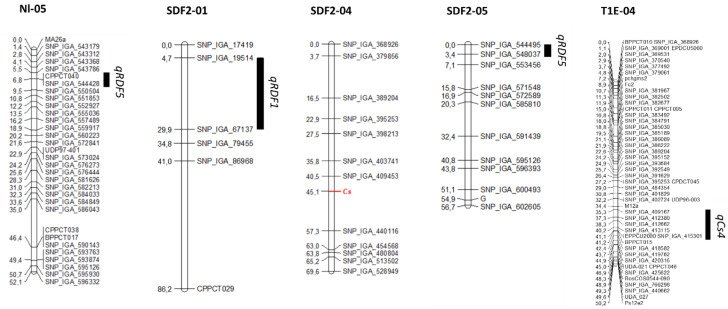
Genetic maps and QTLs from Nectalady (Nl) map of the BtxNl population, and SDF2 and T1E maps. *Cs* is indicated in red. Black bars indicate the LOD-1 interval.

The RDF trait has been studied in two segregating populations. In the peach cross Belbinette × Nectalady (Bb × Nl), we obtained data for RDF in 74 individuals. The remaining 24 had the slow-ripening (SR) phenotype, resulting in fruit that did not mature; therefore, RDF could not be scored. Thirty-seven individuals presented RDF and 37 did not. The observed segregation agrees with a 1:1 segregation (χ^2^ = 0), suggesting that the trait could be controlled by a single gene. When mapping this trait as monogenic, we did not find any position in the genetic map where all the individuals fitted. Using QTL analysis, a major QTL (qRDF5) with an LOD score of 16,19 and explaining a 63,5% of the phenotypic variance was detected at the beginning of G5, between markers SNP_IGA_543786 and SNP_IGA_550504, located at positions 467.067 and 2.041.358 bp of the *P. persica* v2.0 genome respectively (Figure 3).

In the selfed progeny of SweetDream (SDF2), we obtained data from 193 individuals. For the other 225 individuals, 83 were SR and 142 had no fruit. A total of 105 were RDF, while the remaining 88 were not. That also agreed with a 1:1 segregation (χ^2^ = 1.50). Mapping as a monogenic trait was unsuccessful, so we studied the trait as quantitative. In this case, two significant QTLs were identified: the first, *qRDF5*, at the beginning of G5, with an LOD score of 4.96 and explaining a 11.2% of the phenotypic variance and flanked by markers SNP_IGA_544495 and SNP_IGA_548037, corresponding to the physical positions 610,569 and 1,376,475 bp, respectively; the second, *qRDF1*, at the beginning of G1, with an LOD score of 4.77 and explaining 10.8% of the phenotypic variance. It was flanked by markers SNP_IGA_19514 and SNP_IGA_67137, positioned at 6,872,054 and 20,219,128 bp, respectively.

**Figure 4:**
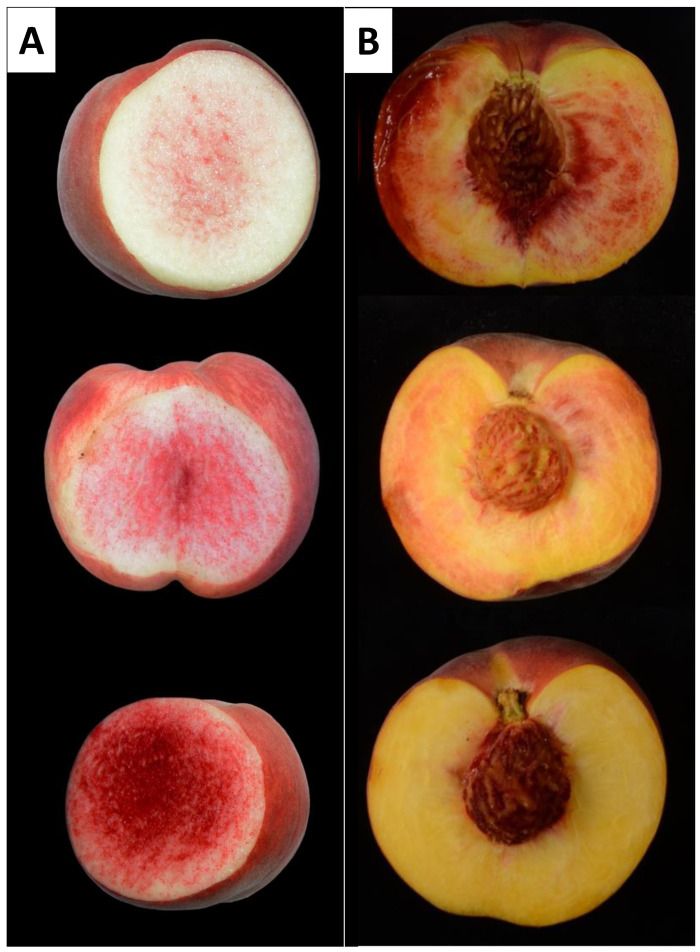
Images of three different individuals from the BbxNl (**A**) and SDF2 (**B**) populations showing the different intensities of the RDF phenotype.

In both populations, the amount of RDF varied considerably, with some individuals presenting fruit flesh with a few red dots, while in others the red dots almost covered the whole mesocarp (Figure 4).

### 2.3. QTL Analysis of Cs in SDF2 and in T1E

The CAS trait was studied in two segregating populations, one intraspecific (SDF2) and the other interspecific (the progeny of the hybrid of Texas almond x Earlygold peach, backcrossed with Earlygold). The SDF2 intraspecific population was evaluated in 2021. Of the 193 individuals evaluated, 133 had red flesh around the stone (68.91%) and 60 individuals had yellow flesh around the stone (31.09%). The segregation observed fitted a 3:1 ratio (χ^2^ = 3.82), suggesting that the trait was controlled by a single gene with dominant inheritance, that was mapped between markers SNP_IGA_409453 and SNP_IGA_440116, which correspond to the physical position spanning from positions 10.396.616 to 16.084.694 bp, respectively (Figure 3).

**Figure 5:**
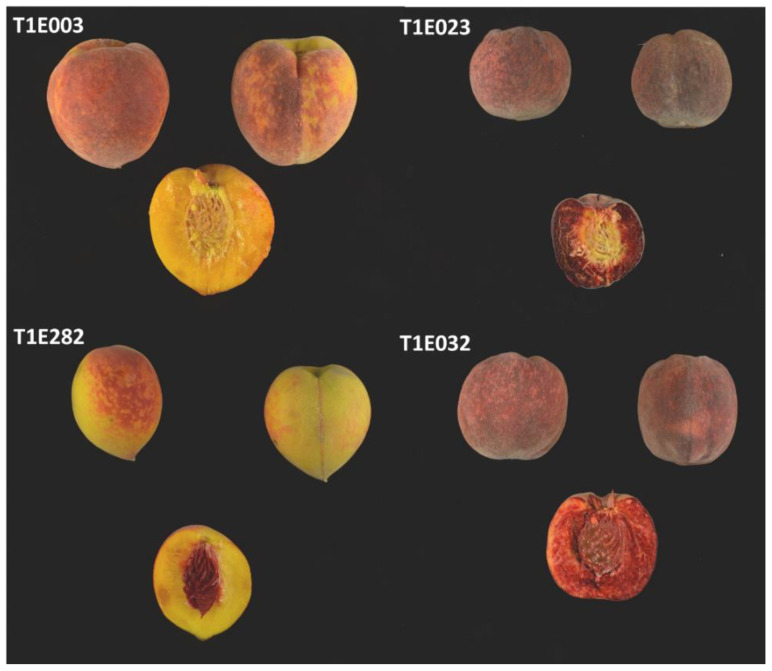
Images of different fruit of different T1E individuals with yellow flesh and yellow CAS (T1E003), red flesh and yellow CAS (T1E023), yellow flesh and red CAS (T1E282) and red flesh and red CAS (T1E032).

The T1E population was evaluated for CAS for 3 years (2017, 2018 and 2019). Fruit showing different combinations of CAS and red flesh can be observed in Figure 5. In some cases, the determination of the CAS trait was difficult; therefore, individuals showing inconsistencies during the three years were discarded from the analysis. Finally, we used 125 individuals showing consistent data during all the years that were evaluated. Fifty-five presented no CAS and 70 were red. Using these data, we identified a QTL in G4, *qCs4*, with a LOD score of 7.59 and explaining a 24.4% of the phenotypic variance. This QTL was located between markers SNP_IGA_409167 and SNP_IGA_419762, which corresponds to the physical position between 10,363,342 and 13,675,935 bp in the *Prunus* reference genome v2.0 [19].

## 3. Discussion

### 3.1. Peach Red Flesh Patterns

The characterization of the red flesh patterns in the IRTA’s peach commercial cultivar collection indicates that beyond the main peach red flesh patterns already described in the bibliography and of known genetic basis (*Bf*, *DBF*, *DBF2* and *Cs* genes), there are other color patterns that may be affected by the same or different genes that are usually not considered for commercial purposes despite their high frequency in peach cultivars. The information provided here could allow peach breeders and germplasm curators to have a more comprehensive understanding of the different red flesh patterns observed in peach flesh. This is also important, as these characteristics are linked to important nutritional and health-promoting properties, at a moment when breeding programs are starting to release new peach cultivars with red flesh.

### 3.2. Relationship between RDF and DBF in Peach

RDF is a trait that is easy to observe and is present in almost half of the new commercial peach cultivars in the IRTA’s peach commercial cultivar collection, which is a representation of the main peach breeding programs in the world. As far as we know, the genetic inheritance of this trait has not been described before. This trait has been mapped in two different segregating populations as a major QTL called qRDF5 at the beginning of G5 in a region between 0.6 and 1.4 Mb of the *Prunus* reference genome v2.0. A very important gene related to red flesh that has been previously described in this region is the *DBF* gene identified in the Wu Yue Xian red-fleshed cultivar [14]. In the same region, another gene called BLOOD (BL) was described in the Dahongpao red-fleshed cultivar [20]. Using fine mapping and expression analysis, an NAC transcription factor (*BL*) was proposed as the causal gene. This gene, annotated in the *Prunus* reference genome v2.0 as *Prupe.5G006200*, is in the position 709,695 bp. Zhou et al. [20] also showed that the heterodimer of *BL* and *PpNAC1*, another gene encoding for an NAC transcription factor that is a strong candidate for a major peach gene controlling maturity date (*MD*), can activate the transcription of *PpMYB10.1*, resulting in anthocyanin pigmentation in tobacco. *MYB10.1* has been described as a gene responsible for several traits related to anthocyanin biosynthesis in peach as CAS or red skin [21,22]. Therefore, a possible explanation is that qRDF5 is produced by a different allele of *DBF*. We checked in the IRTA’s peach commercial cultivar collection to see if there was a correlation between the presence of RDF and MD, but we did not find any.

We also want to highlight that 0.3 Mb away from the *BL* gene, there is a marker highly associated with the subacid gene (*D*). This gene is also segregating in both Bb x Nl and SDF2 populations. As has been observed in crab apples [23] pH can affect anthocyanin stability. That, together with the fact that RDF is only expressed at the last stages of fruit development and increased when fruits were overripened, could explain in part why in some individuals there is a faint expression of the RDF while in others is more intense. Furthermore, the linkage between the genes *BL* and *D* could have implications for peach breeding, making it more difficult to obtain varieties with red flesh and subacid taste.

In the SDF2 population, in addition to the major gene in G5, we identified another QTL at the top of G1 between markers SNP_IGA_19514 and SNP_IGA_67137 (6.872.054 and 20.219.128 pb). In a GWAS study with a panel of 129 Chinese accessions, this same genomic region (between 11,7 and 13.1 Mbp) was associated with anthocyanin content codified as presence or absence, but not as a quantitative trait [24]. A QTL was also reported in this region in a BC2 population derived from an interspecific cross between peach and *P. davidiana* [25]. The presence of other minor QTLs affecting RDF could be another factor explaining the difference in intensity observed for RDF.

### 3.3. Relationship between Cs and MD in Peach

The *Cs* gene was first mapped in G3 using a segregating population [16,17]. Later, using association analysis, its location in G3 was confirmed and a 487 bp deletion affecting the PpMYB10.1 promoter region was found to be associated with the trait [18], although it was not predictive in many accessions. In our case, and using two different segregating populations, we identified a gene in the region of G4 where a major gene for maturity date (*MD*) is located [18,26], this region being a hotspot for genes of interest in various *Prunus* species [4]. In the IRTA’s peach commercial cultivar collection, we also observed that CAS was only found in late cultivars, suggesting an interaction between CAS and MD. A possible hypothesis is that individuals with CAS need to have the appropriate alleles for MYB10.1 (probably with the deletion of the 487 bp fragment described by [18]) and be medium or late ripening (probably carrying some specific alleles in the MD locus from G4). A similar situation, where an interaction between the G3 and G4 regions where *MYB10.1* and *MD* loci are located, has already been described for the red leaves at senescence [27]. In that case, individuals with red leaves at senescence carry the appropriate alleles at both loci, meaning that all individuals with red leaves at senescence are early ripening, but not all early ripening individuals have the red leaves at senescence [27]. In apple, it an interaction between red flesh and maturity date has also been observed. In this species, two types of red flesh have been described [28,29]. The first type displays anthocyanin pigmentation in all tissue, including fruit and foliage, and is controlled by a tandem duplication of a 23 bp motif containing a MYB-binding site in the promoter region of MYB10 [30]. In type 2, the phenotype is characterized by the accumulation of anthocyanin in the fruit cortex only in late maturity, although the responsible mutation remains unclear [29].

### 3.4. Red Dots under the Skin (RDS)

The other trait that we measured we called RDS. We observed than in some cases, the phenotype was very clear, while in other the intensity of red dots was very light or the reddish color could not be observed in the whole fruit, but only in one side or in the lower part of the fruit around the tip. In addition, in some of the cultivars where the presence of the RDS was clearer and more uniform, it could be that there are cultivars with RDF, but with no red around the stone, therefore looking like if the red dots were only present under the skin. Therefore, we propose to further characterize this phenotype to discard that what we called RDS here is not RDF with no CAS, or to look for segregating populations where this phenotype could be further studied. A potential parental for these populations would be Britney Lane, because in this case, the phenotype was clearer and more uniform than in all the other cultivars.

## 4. Conclusions

The results obtained here allow better understanding of peach flesh anthocyanin patterns and paves the way to develop an integrated molecular marker system that would allow selecting for specific peach red flesh patterns. In the case of RDF, we identified a QTL at the beginning of G5 called qRDF5 and we hypothesized that it could be produced by an allele of the DBF or BL gene. We also highlighted the importance of the close linkage between all these genes and the subacid gene (D), because it could be important to develop subacid cultivars with red flesh produced by these genes. For CAS, we identified the gene producing this trait in a different region of G4, in contrast to all previous information reporting *Cs* in G3. Our hypothesis is that in some plant material with the Cs allele from G3 producing CAS, the trait is not expressed because of its interaction with the allele from MD in G4 controlling the maturity date. Finally, we proposed that some work should be done with RDS to understand if there are other genes controlling this trait or if it is a phenotype produced by the interaction of RDF and CAS.

## 5. Materials and Methods

### 5.1. Plant Material

A commercial peach cultivar collection and three biparental segregating populations were used in this study. The IRTA commercial cultivar collection consists of 159 cultivars representing a wide range of breeding programs and including different peach typologies as peaches and nectarines, of round and flat shape, melting and non-melting flesh, and white, yellow and red flesh. The first segregating population used was an F_1_ segregating population called Bb × Nl, comprising 98 individuals from the cross between Belbinette and Nectalady [31,32]. The second population was an F_2_ segregating population of 431 individuals, called SDF2, that was derived from the selfing of Sweet Dream [33]. The third one was an interspecific BC_1_ segregating population of 190 individuals called T1E that was generated by backcrossing the F_1_ hybrid between the Texas almond cultivar and the Earlygold peach cultivar, and backcrossed to Earlygold (Donoso et al. 2016). The IRTA cultivar collection was grafted in GF677 rootstock and the Bb × Nl population was grafted on Cadaman rootstock. The SDF2 population was on their own roots and the T1E population was grafted on Garnem rootstock. All the trees were planted in the fields at the IRTA Experimental Station at Gimenells (Lleida, Spain) and managed according to standard agronomic practices. The climate was cold semiarid Mediterranean (Bsk, according to the Köppen–Geiger climate classification system), with annual precipitation of 350 mm, and a mean summer daily temperature of 32 °C.

### 5.2. Phenotyping

Five peaches from each tree were collected in the orchard at the commercial maturity stage, and maturity date (MD) was scored in Julian days. Commercial maturity harvest date was determined based on the visual background color change of the fruit skin and/or flesh firmness ranging from 39 to 49 N. Fruit flesh color was measured in the IRTA’s peach commercial cultivar collection in 2019. The Bb × Nl population was measured in 2011, SDF2 in 2021 and T1E in 2017, 2018 and 2019. As different color patterns were observed, we measured four separate traits: flesh color (FC) as white, yellow or red, presence or absence of red dots in the flesh (RDF), presence or absence of red dots under the skin (RDS) and presence or absence of red color around the stone (CAS). Intensity or percentage of RDF was not scored because we observed that the intensity was slightly evolving during ripening.

### 5.3. QTL Analysis

For QTL analysis, we used the genetic maps already available for the three segregating populations. For the Bb × Nl F_1_ population, a genetic map was already available [31]. The genotypes obtained after genotyping the population with the 9 K International Peach SNP Consortium array [32] were added to the map using Joinmap5.1 [34]. A genetic map was obtained for each parental line—Bb and Nl. Grouping was performed with a threshold of LOD ≥ 3.0 and genetic distance was calculated with the Kosambi function. Linkage group terminology was according to the *Prunus* reference genome v2.0 [19]. The second map used was for the SDF2 population already described by Eduardo et al. [33]. We added three new SSRs in the regions not covered with an initial set of 64 SNPs. Finally, we used the T1E genetic maps described in Donoso et al. [15] for each parental of the T1E population, F1 hybrid and Earlygold (E). They had 2031 and 1091 markers and covered a genetic distance of 370.1 and 520.4 cM, respectively.

QTLs were analyzed using the interval mapping method with MapQTL 6.0 software [35]. QTLs with an LOD score greater than or equal to 3.0 were considered significant. Maps and QTL positions were drawn using the MapChart 2.1 software [36].

## Data Availability

Data are contained within the article or supplementary material. Data not included in the manuscript are available on request from the corresponding author.

## Supplementary Materials

The following supporting information can be downloaded at https://www.mdpi.com/article/10.3390/plants12020394/s1. Table S1: List of the 159 peach cultivars evaluated and phenotypic information for the traits evaluated: flesh color (FC), red dots in the flesh (RDF), red dots under the skin (RDS), red color around the stone (CAS), and maturity date 2019 (MD).
