# Supplementary material for: Inheritance of Fruit Red-Flesh Patterns in Peach
Source: Plants (Basel). 2023 Jan 14;12(2):394. doi: 10.3390/plants12020394 (PMC9862646; doi:10.3390/plants12020394)
Supplement: Supplementary file 1 [file plants-12-00394-s001.zip › plants-2146014-supplementary.pdf]

Plant name	Flesh color (FC)	Red dots in the flesh (RDF)	Red dots under the skin (RDS)	Red color around the stone (Cs)	MD 2019
ALABAMA	W	no	si	no	196
ALBATINE	W	si	no	no	176
ALCATRAZ	W	no	no	no	171
ALMAMIEL	Y	no	no	si	220
ALMANEBO	Y	no	no	no	169
ALMANOR	Y	si	no	no	213
ALMONTIEL	Y	no	no	no	189
AMADEUS	W	no	si	no	182
AMBRA	Y	si	no	no	182
ARGOS	Y	no	no	no	171
ARIZONA	Y	si	no	no	192
ARTEMIS	Y	si	no	no	164
ASF 10-36	W	no	no	si	267
ASF 10-53	W	no	no	si	267
ASTORIA	Y	si	no	no	154
AUTUMN FLAME 38	Y	no	no	si	267
BEAUTY PEARL	W	no	si	no	189
BIG TOP	Y	si	no	no	182
BOREAL	W	no	no	no	169
BORNEO	Y	no	no	no	171
BRITNEY LANE	Y	si	si	no	171
BUENOS	Y	no	no	no	185
BUENOS II	Y	no	no	no	185
BURMA	W	si	no	si	224
CAKEREVE	W	si	no	no	189
CANDY PEARL	W	no	no	no	189
CARACAS	Y	si	no	no	189
CARAMBA	W	no	no	no	196
CARLA	Y	si	no	no	161
CATHERINA	Y	no	no	no	196
CLARISS	Y	si	no	no	210
CONTESSA	W	no	no	no	169
CRISPDIVA	Y	no	no	si	252
CRISPDREAM	Y	si	no	no	217
CRISPGOUDE	Y	si	no	no	213
CRISPSOL	Y	si	no	no	245
CRISPSREVE	Y	si	no	no	203
CRISPTOLAM	Y	si	no	no	169
DIABLOTINA	R	-	-	-	224
DIAMOND PEARL	W	si	no	no	176
E45.004	W	no	no	no	171
ELEGANT LADY	Y	no	no	si	224
EMERAUDE	W	si	si	no	196
ESMERALDA	Y	no	no	si	252
EXTREME 618	Y	no	no	si	259
FEBE	Y	no	no	si	239
FERCLUSE	Y	no	no	no	217
FERLATE	Y	no	no	no	252
FERLOT	Y	no	no	no	232
FILOE	W	si	no	no	224
FLATBUZZ	W	si	no	no	182
FLATCANDY	W	si	si	no	182
FLATCHIEF	W	no	si	no	199
FLATDIVA	W	no	no	si	255

Plant name	Flesh color (FC)	Red dots in the flesh (RDF)	Red dots under the skin (RDS)	Red color around the stone (Cs)	MD 2019
FLATELSE	W	si	si	no	206
FLATSTAR	W	no	no	si	239
FLATWO	W	si	no	no	164
GARCICA	W	si	no	no	189
GARDETA	Y	si	no	no	182
GEA	Y	no	no	no	203
GLADYS	W	no	no	si	259
GRAND CANDY	W	no	no	no	185
GUAYOX 4-24	W	si	no	no	224
GUAYOX36	W	si	no	no	245
IBIZA	W	no	no	no	213
ICE PEACH	W	no	no	no	239
IVORY NIGHT	W	no	no	si	252
IVORY STAR	W	si	no	no	199
IVORY SUN	W	no	no	si	252
KINOLEA	Y	si	no	no	239
LAMBADA	Y	si	no	no	210
LEA	W	si	no	no	176
LEONIDA	W	si	no	no	161
LUCIANA	Y	si	no	no	213
LUCIUS	W	no	no	si	252
MAREVA	W	si	no	no	171
MELISSA	W	si	no	no	220
MELOX26	W	si	no	no	179
MELOX31	W	no	no	no	226
MELOX34	W	si	no	si	239
MELOX37	W	no	no	si	259
MISTRAL30	Y	no	no	no	185
MOMBASSA	Y	no	si	no	189
MONANGE	W	si	no	no	189
MONATUM	W	no	no	no	157
MONIFIC	Y	si	si	no	171
MONMEIL	W	no	no	no	199
MONSOLE	W	si	no	no	171
MONTICA	Y	no	no	no	203
NABEPI	W	si	no	no	179
NABIDELICE	W	no	si	no	192
NAJATAR	Y	no	no	si	245
NAJICAN	Y	si	no	no	217
NB895	W	no	no	si	259
NECTABINGO	Y	si	no	no	185
NECTADIVA	Y	no	no	si	255
NECTAFLASH	Y	no	no	no	169
NECTAKING	Y	no	si	no	203
NECTANA	Y	no	no	si	245
NECTAPERF	W	no	no	si	245
NECTARANGELS	W	no	no	no	206
NECTARELSE	W	no	no	si	267
NECTARNOVALA	W	no	no	no	179
NECTARNOW	W	si	no	no	203
NECTARONDA	Y	si	no	no	192
NECTATINTO	Y	no	no	si	259
NETIX 31	Y	si	no	no	217
NETIX22-57	Y	no	no	si	239

Plant name	Flesh color (FC)	Red dots in the flesh (RDF)	Red dots under the skin (RDS)	Red color around the stone (Cs)	MD 2019
NETIX25	Y	no	no	no	171
NETIX28	Y	no	si	no	199
NETIX30	Y	si	no	si	213
NIRVANA	W	si	no	no	199
NORACILA	Y	no	no	no	171
O'HENRY	Y	si	no	si	245
OPERA	W	no	no	si	224
ORENGA	W	si	si	no	196
OSIRIS	Y	no	no	no	164
OSIRIS	Y	no	no	no	164
PABEARLY	W	no	no	no	164
PAJATO	Y	no	no	no	157
PAMELA	W	si	no	no	164
PAMPANA	Y	si	no	no	164
PATTY	W	no	si	no	182
PLAGOLD 19S	Y	no	si	no	182
PLATIMED	W	no	no	no	185
PRINCE PEARL	W	no	no	no	161
REDIX120	W	si	si	no	199
REDIX24	W	si	no	no	164
REDIX30	W	si	no	no	210
RMC 3-11-1-7-4	Y	si	no	no	182
ROMEA	Y	no	no	no	185
ROYAL SUMMER	Y	no	no	no	199
SAMANTHA	W	si	no	no	164
SMOOTY	Y	no	no	no	164
SNOW FLAKE	W	no	no	no	164
SNOW FLAME 19	W	no	no	no	154
SNOW FLAME 23	W	no	no	no	164
SNOW FLAME 36	W	no	no	si	259
SNOW FLAME 38	W	no	no	si	267
SNOW LADY	W	si	si	no	232
SPRING FLAME 20	Y	no	no	no	161
SUGAR TIME	Y	no	no	no	164
SUMMER FLAME 34	Y	no	no	si	232
SUMMER FLARE 36	Y	no	no	si	259
SUN BRIGHT	Y	si	no	si	239
SWEET HENRY	Y	no	no	si	252
SWEET REGAL	W	no	si	no	210
SWEETALY	W	no	no	no	169
SWEETBANG	W	si	si	no	182
SWEETCAP	W	si	no	no	220
SWEETDREAM	Y	si	no	no	224
TANGOS II	W	no	no	no	199
TIETAR	Y	no	no	no	267
TIFANY	W	si	no	no	213
TOP PEARL	W	no	no	no	189
VENUS	Y	si	no	no	224
YELLOW STONE	Y	no	no	no	179
ZINCAL 18S	Y	si	no	no	182
ZINCAL 25S	Y	no	si	no	185
